# Antibacterial Properties of *Aloe vera* on Intracanal Medicaments against *Enterococcus faecalis* Biofilm at Different Stages of Development

**DOI:** 10.1155/2020/8855277

**Published:** 2020-12-28

**Authors:** Negin Ghasemi, Mahsa Behnezhad, Mohammad Asgharzadeh, Elham Zeinalzadeh, Hossein Samadi Kafil

**Affiliations:** ^1^Research Center for Pharmaceutical Nanotechnology, Department of Endodontics, Tabriz University of Medical Sciences, Tabriz, Iran; ^2^Dental and Periodontal Research Center, Tabriz University of Medical Sciences, Tabriz, Iran; ^3^Biotechnology Research Center, Tabriz University of Medical Sciences, Tabriz, Iran; ^4^Hematology and Oncology Research Center, Tabriz University of Medical Sciences, Tabriz, Iran; ^5^Drug Applied Research Center, Tabriz University of Medical Sciences, Tabriz, Iran

## Abstract

**Background:**

Use of herbal compounds as an intracanal medicament in the field of endodontics has become noteworthy, one of which is the *Aloe vera* compound whose antibacterial effect has already been proven in the planktonic form of *Enterococcus faecalis*. The purpose of this study is to evaluate the antibacterial effect of *Aloe vera* on *E. faecalis* biofilms at the 4^th^ and 6^th^ week of development.

**Materials and Methods:**

130 single root canal teeth without anomalies and caries were used. They were divided into two groups of 65 teeth for four and six weeks of biofilm production. Five samples of each group were examined for confirmation of biofilm formation under an electron microscope. Study groups were investigated with an antimicrobial agent as an intracanal medicament including 20 samples treated with *Aloe vera*, calcium hydroxide, and phosphate-buffered saline, and biofilm and survival of pathogens were investigated. Dentin chip suspensions were used for colony-forming unit (CFU) counting to estimate remaining *E. faecalis* counts.

**Results:**

The CFU mean in the 4^th^ week subgroup in *Aloe vera*, phosphate-buffered saline, and calcium hydroxide was 0, 69166.66 ± 31688.58, and 25000 ± 30822.07, and in the 6^th^ week, it was 136.36 ± 323.33, 95000 ± 12247.44, and 27501.66 ± 36570.34, respectively, which showed a significant difference between the used materials (*p* < 0.05).

**Conclusion:**

*Aloe vera*, in contrast to calcium hydroxide, eliminated 4^th^ and 6^th^ week biofilms and showed remarkable antibacterial properties against *E. faecalis* biofilm. These results support potency of *Aloe vera* to use as a natural antimicrobial material in the intracanal medicament.

## 1. Introduction

Despite the application of various canal cleaning techniques and tools, as well as washing solutions and antimicrobial drugs, resistant microorganisms such as *Enterococcus faecalis* are still a challenge to the success of root canal therapy [1–3]. This bacterium is the most common microorganism in the pathogenicity of recurrent infections and resistant endemic infections, which is found in 22–77% of failed cases [4, 5]. It is a Gram-positive facultative anaerobic bacterium which has high resistance to high pH and concentrations of saline solution and has a high ability for biofilm formation, one of the most important virulence factors [2, 6].

The biofilm is a dynamic and complex structure of organisms, causes resistance to environmental stresses such as antimicrobials, and is more difficult than planktonic bacteria to eliminate [7]. Because biofilms make bacteria resistant to phagocytosis, antibodies, and antimicrobial agents, the problem will become more apparent with the passage of time and the advent of mineralization that will lead to biofilm maturation [8, 9].

The use of intracanal medicaments is one of the ways to eliminate or reduce the microbial population. Calcium hydroxide is the most common intracanal medicament, which, despite the appropriate antibacterial spectrum, has little effect on *E. faecalis* [9–11].

Due to the side effects of synthetic drugs, the trend towards the use of herbal medicines has recently been considered [12]. *Aloe vera* is an antibacterial, antiviral, antifungal, and anti-inflammatory herbal compound and is used in toothpaste and mouthwashes. It has also been studied as a canal washing agent [13–16]. This herbal compound has an inhibitory effect on many oral pathogens, including *E. faecalis*, *Streptococcus pyogenes,* and *Candida albicans*, due to the anthraquinone phenolic compounds [13, 17].

*Aloe vera* is a gel and can be used as an intracanal medicament. *Aloe vera* is biocompatible and has no toxicity problem in the long term adjacent to the tissues around the root [16, 18]. Agar diffusion, broth diffusion, and direct contact method are the most conventional methods to evaluate the antibacterial property of such components [13, 15, 17, 19]. The purpose of this study was to evaluate the antibacterial properties of *Aloe vera* gel as an intracanal medicament against *E. faecalis* biofilm at the 4^th^ and 6^th^ week of development by the colony-forming unit (CFU) counting method to see whether *Aloe vera* gel has equal activity with other conventional antimicrobial agents.

## 2. Materials and Methods

### 2.1. Preparation of Samples

This study was approved by the local ethics committee with reference number IR.TBZMED.VCR.REC.1398.401. In this study, 130 single root canal teeth without anomalies and caries were used. Periodontal tissue and plaque of all samples were removed by the ultrasonic method, and the samples were kept in a chloramine T solution of 0.5% until the study was completed. The crown of the teeth was cut off from the cementoenamel junction by a diamond disc with water cooled to a standard length of 12 mm.

After determining the length of the function by file K number 15 (Mani Co., Japan), the cleansing and shaping of the canal were performed by a ProTaper rotary system [20], and the file used to prepare was F5 to prepare the canal. In the next step, 2.5% washing solution was used during the sodium hypochlorite (Dental Sky Wholesaler Ltd., England) procedure, which was performed by a 10 ml syringe with a 30 gauge needle. In order to remove the smear layer, 1 milliliter of hypochlorite solution at 25.5% for one minute and then 1 milliliter of 17% EDTA solution for 1 minute were used, and the final solution was normal saline. Five samples were randomly selected ensuring the cleanliness of the dentin walls and the absence of bacteria, and the openness of dentinal tubules was investigated under the electron microscope. The study samples, according to the age of biofilm formed in the canal, were grouped as follows: Group A: four-week biofilm (20 samples); Group B: six-week biofilm (20 samples).

After the preparation of canals, the samples were autoclaved at 121°C and 15 psi pressure for 20 minutes, in order to kill all of the existing microorganisms. To check the efficiency of the mentioned process, all samples were incubated immediately in Brain Heart Infusion Broth (BHIB) (Merck, Germany) for 24 hours at 37°C. In order to form *E. faecalis* ATCC®29212™ biofilm, pure bacteria culture was incubated in BHIB at 37°C at 10% CO_2_ pressure for 24 hours, and a solution containing bacterial cells of 10^8^ cells/ml, which was equivalent to an optical density of 0.1 by using the UV spectrophotometer, was prepared [21, 22]. The teeth were placed in sterile 1.5 ml Eppendorf vials containing sterile 1 ml of the culture media, and 0.1 ml of the mentioned solution (microbial solution) was added to each vial. Each day, 1 ml of new culture medium (without bacteria) was added to the vial. In the first group, this process was performed for 4 weeks and in the second group for 6 weeks.

**Figure 1:**
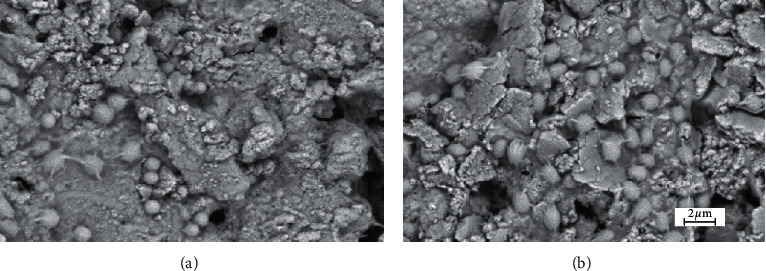
Biofilm formation by *Enterococcus faecalis* in the intracanal after (a) 4^th^ week and (b) 6^th^ week of culture. Photo was captured by SEM microscopy with gold staining of the surface of canals for biofilm confirmation.

After the mentioned time period, 5 samples from each group were randomly selected and examined under an electron microscope to ensure that the biofilm was formed (Figure 1).

According to the antimicrobial agent used, each group was divided into 3 subgroups as follows:

  Subgroup 1 (20 samples for *Aloe vera*): 1 ml *Aloe vera* gel (100% *Aloe vera* gel, Sillaneh Co., Iran) was injected into each canal
  Subgroup 2 (20 samples): calcium hydroxide was placed in the canal using a Lentulo spiral
  Subgroup 3 (PBS) (20 samples): this subgroup is a control group, and the canal is only washed with 2 millimeters of this substance

After 1 week incubation at 37^º^C in an incubator with normal humidity, antimicrobial agents were washed out of the canals with 10 ml of distilled water and then dried with the paper point. To collect dental debris, a 4-5 Gates Glidden drill was used with a class II biological hood and then was transmitted to a microcentrifuge containing 1 ml of sterile TSB medium and was incubated in a microaerophilic condition at 37°C for 24 hours. After the completion of the microaerophilic condition incubation cycle, the contents of each tube in a culture medium after 24 hours of incubation were diluted in a 3-time-diluted physiologic serum (10 *μ*l of bacterial culture medium and 90 *μ*l of fresh medium) and then cultured on a specific plate (Mueller–Hinton agar, Merck, Germany). The number of bacteria was determined by the colony count machine.

### 2.2. Statistical Analysis

Results of the study were reported using descriptive statistics (mean ± standard deviation). Kruskal–Wallis test was used to compare CFU levels in three groups: *Aloe vera*, phosphate-buffered saline (PBS), and calcium hydroxide at 4^th^ and 6^th^ week intervals, due to the unusual distribution of data. The comparison between CFU at 4^th^ and 6^th^ weeks separately in each group, considering the abnormal distribution of data, was performed by Mann–Whitney *U* test. Normality of data was analyzed by Kolmogorov–Smirnov test. Statistical analysis was performed using SPSS17 software, and *p* < 0.05 was considered as significant.

## 3. Results

Results of descriptive statistics are presented in Table 1. The results that are presented in Table 1 showed no statistically significant difference between the 4- and 6-week levels in each group in comparison with themselves (*p* value >0.05).

Kruskal–Wallis comparison between the mean values of CFU of bacteria among the groups represented that there was a significant difference among the three groups with the other group in the 4^th^ week and 6^th^ week after the exposure of antimicrobial agents and biofilm (*p* value <0.001) (Table 1).

## 4. Discussion

The aim of this study was to investigate the antibacterial effect of *Aloe vera* gel on 4^th^ and 6^th^ weeks of *E. faecalis* biofilm and to compare it with calcium hydroxide. The results of the study showed a significant positive effect of *Aloe vera* on the elimination of biofilms at both biofilm development stages; in contrast, calcium hydroxide represented a very weak antibacterial effect. It was in agreement with our null hypothesis, and *Aloe vera* had acceptable efficacy against microbial colonization. *E. faecalis* is one of the main agents related to intracanal infections from a polymicrobial environment of the oral cavity.

In order to have successful treatment of the root, removal of microorganisms from the canal is essential. Microorganisms are present in two forms of biofilm and planktonic inside the canal, and eliminating biofilm is far more difficult than the planktonic state [23, 24].

*E. faecalis* is the most common microorganism in failed cases in treatment and resistant infections [25, 26]. This bacterium has a high potential to invade dentin tubules and form biofilms, so it is resistant to the conditions created by the instrument in the canal, the use of cleansing agents, and the use of intracanal medication, such as calcium hydroxide [27]. In this study, *E. faecalis* bacterium was used that has a high prevalence in resistant infections, and it is difficult to eliminate.

Unlike most antimicrobial studies, we used biofilms instead of the planktonic form, which are 1000 times more resistant than the planktonic state. Biofilm is a dynamic structure of buried bacteria in a polysaccharide matrix [28]. According to Kishen et al.'s [23] study, 4^th^ week after incubation, the bacteria cover all surfaces of the dentin, and after the 6^th^ week, they become mature with the advent of mineralization [23, 29, 30]. As the age of the biofilm increases, its resistance increases due to calcification and organization. Selected periods of this study were 4^th^ and 6^th^ weeks [8].

In order to reconstruct the clinical condition, extracted teeth were used, and biofilm was cultured in the teeth, and within the canal, antimicrobial agents were exposed to the effect of dentin blockage on the antimicrobial ability of the materials used.

The antimicrobial agents used in this study were calcium hydroxide and *Aloe vera*. Calcium hydroxide is the gold standard of intracanal medicament [31]. These materials can damage the cell wall of the bacteria or cause denaturation of proteins and DNA which causes bacterial death [32, 33].

*E. faecalis* is relatively resistant to most of the antimicrobial agents such as calcium hydroxide (by bacterial efflux pump activity) [34]. In this study, the result is also consistent with the results of other studies on the disability of calcium hydroxide. However, as previously mentioned in this study, biofilms have been used instead of the planktonic state which increases the value of this investigation. Investigation method of antimicrobial activity was CFU counting. In previous studies, the method used was mostly agar diffusion, which was less accurate than CFU [35, 36].

Recently, attention has been drawn to the use of herbal medicines in endodontics because synthetic drugs with paregoric-like toxicity increase bacterial resistance. Instead, herbal medicines are cheap, available, and less toxic, have therapeutic properties, and are well received by the patient [34, 37].

The herbal medicine used in this study was *Aloe vera*, which is herbal combination with anti-inflammatory, antibacterial, antifungal, wound healing, and pain relief properties and is used in various dental areas [17, 37]. In the present study, its antibacterial properties are better than those of calcium hydroxide. *Aloe vera* has a strong antibacterial effect in comparison to calcium hydroxide as an antioxidant and reduces free radical production. On the contrary, its cytotoxicity is lower than calcium hydroxide [11, 15, 17, 34, 38]. This herb can grow in wild and wide ranges of environments. It can survive in environments consisting several risk factors; therefore, it should produce several antibacterial agents to survive.

*Aloe vera* stimulates fibroblast growth and synthesis of collagen. Stimulation of pulp cell proliferation, differentiation, and extracellular matrix mineralization are other properties of *Aloe vera* [16, 18, 39, 40]. Considering that *Aloe vera* has an analgesic effect [41], it can be used as a lubricant and smooth dressing during the preparation of the canal. However, its effect on the physical properties of the dentin, such as microhardness, and its effect on resistance to root fracture in cases where it is used as the intracanal medicament are better to be surveyed.

## 5. Conclusion

By the results obtained in this study and previous descriptions on *Aloe vera*, it had acceptable antibiofilm activity as a candidate for dental treatment process. Our results showed *Aloe vera* can inhibit the growth of *E. faecalis* equal with common antimicrobial agents. However, more studies for its synergy with other conventional therapies are recommended.

## Figures and Tables

**Table 1:** Mean ± SD of the colony-forming unit of *Enterococcus faecalis* after treatment with different antimicrobial agents.

Group	Time^*∗*^	*N* ^∗∗^	Min.^∗∗∗^	Max.^∗∗∗∗^	Mean ± standard deviation CFU^∗∗∗∗∗^
*Aloe vera*	4 weeks	20	0	0	0
	6 weeks	20	0	1000	136.36 ± 323.33

Phosphate-buffered saline (PBS)	4 weeks	20	20000	100000	69166.66 ± 31688.58
	6 weeks	20	20000	70000	95000 ± 12247.44

Calcium hydroxide (CaOH_2_)	4 weeks	20	0	80000	25000 ± 30822.07
	6 weeks	20	10	100000	27501.66 ± 36570.34

^∗^Time of culture for biofilm formation in the dental root area, ^∗∗^number of samples which were used for the study, ^∗∗∗^lowest number of colony formation after exposure with groups' examination solution, ^∗∗∗∗^highest number of colony formation after exposure with groups' examination solution, and ^∗∗∗∗^mean ± standard deviation of colony-forming units.

## Data Availability

The data used to support the findings of this study are included within the article and are available by email to the corresponding author.
