# Corrigendum to “Antibacterial Properties of *Aloe vera* on Intracanal Medicaments against *Enterococcus faecalis* Biofilm at Different Stages of Development”

**DOI:** 10.1155/2021/9825216

**Published:** 2021-06-10

**Authors:** Negin Ghasemi, Mahsa Behnezhad, Mohammad Asgharzadeh, Elham Zeinalzadeh, Hossein Samadi Kafil

**Affiliations:** ^1^Dental and Periodontal Research Center, Tabriz University of Medical Sciences, Tabriz, Iran; ^2^Research Center for Pharmaceutical Nanotechnology, Department of Endodontics, Tabriz University of Medical Sciences, Tabriz, Iran; ^3^Biotechnology Research Center, Tabriz University of Medical Sciences, Tabriz, Iran; ^4^Hematology and Oncology Research Center, Tabriz University of Medical Sciences, Tabriz, Iran; ^5^Drug Applied Research Center, Tabriz University of Medical Sciences, Tabriz, Iran

In the article titled “Antibacterial Properties of *Aloe vera* on Intracanal Medicaments against *Enterococcus faecalis* Biofilm at Different Stages of Development” [1], author Negin Ghasemi was affiliated to “Research Center for Pharmaceutical Nanotechnology, Department of Endodontics, Tabriz University of Medical Sciences, Tabriz, Iran,” which is incorrect and author Mahsa Behnezhad was affiliated to “Dental and Periodontal Research Center, Tabriz University of Medical Sciences, Tabriz, Iran,” which is incorrect.

The correct affiliation for author “Negin Ghasemi” is “Dental and Periodontal Research Center, Tabriz University of Medical Sciences, Tabriz, Iran,” and the correct affiliation for author “Mahsa Behnezhad” is “Research Center for Pharmaceutical Nanotechnology, Department of Endodontics, Tabriz University of Medical Sciences, Tabriz, Iran.”

The corrected list of affiliations is shown in the author information above.
